# 50 years experience with Dupuytren's contracture in the Erlangen University Hospital – A retrospective analysis of 2919 operated hands from 1956 to 2006

**DOI:** 10.1186/1471-2474-8-60

**Published:** 2007-07-04

**Authors:** Bernd Loos, Valerij Puschkin, Raymund E Horch

**Affiliations:** 1 Department of Plastic and Hand Surgery, University Hospital Erlangen, Germany

## Abstract

**Background:**

Dupuytren's disease (DD) is a hand disorder mainly among the northern population. In contrast it is rare in the mediterranean population. Therefore typical habits and dietetic influences have been discussed as well as genetic predisposition. Still, since the first description by Dupuytren in 1834 only little is known about the etiology and pathogenesis of this disease. Some hints were found for a higher prevalence among people with diabetes, alcohol abuse or smoking. Also, intensive manual work or hand injuries have been discussed to have an influence on DD. To our knowledge this is the largest retrospectively evaluated series of symptomatic patients published to date. The study includes patients from the last 50 years. It was performed to show possible correlations between DD and typical risk factors such as diabetes, alcohol consumption, and smoking.

**Methods:**

We retrospectively analysed all patient records with DD documented between 1956 and 2006 in the Surgical University Hospital in Erlangen. Data acquisition was conducted by reviewing the medical records from 1956 to 2006 including data from all patients who were surgically treated because of DD.

**Results:**

We reviewed 2579 male and 340 female surgically treated patients with DD. More than 80% of the patients were between 40 and 70 years old. In 28.9% only the right hand was effected by DD, in 25.3% only the left hand and in 45.8% both hands. In 10.3% of all Patients suffered from Diabetes mellitus. Statistical analysis revealed no significant correlation between diabetes, alcoholism or smoking on the degree of DD in our patients.

**Conclusion:**

Most data are consistent with previously published results from smaller, comparable retrospective studies with regard to right- or left handedness. We could not confirm a statistically significant correlation of DD with diabetes mellitus, severe alcohol consumption, heavy smoking or epilepsy and the stage of the disease as described in other studies. However, in the whole cohort of our operated patients during the last 50 years the prevalence of the above mentioned risk factors is slightly higher than in the normal population.

## Background

Dupuytren's disease (DD) belongs to the group of fibromatoses and affects the hand's palmar fascia. DD is related to the plantar fibromatosis, penile fibromatosis, and fibromatosis of the dorsum of the proximal interphalangeal joints called knuckle pads [1]. Typically, it is characterized by cords and nodules in the palm of the hand, the pathologic counterpart to the tendon and pretendinous bands [2-4]. At the beginning of the disease nodule-formation in the palm of the hand is common. Later nodules may form near the MCP joint or next to the PIP joint of the thumb and digits. Depending on the progress of the individual disease contractures might form along normal fascia structures [3,4]. The disease is generally attributed to the French anatomist and surgeon Baron Guilleaume Dupuytren because of his expertise on the pathogenesis, prognosis, clinical findings, and his description of the treatment of the disease in 1834 [2].

It has been generally agreed that DD is more common in people of the white northern European population (Caucasians), especially from Celtic descent [5-14]. With the inclusion of close relatives the incidence in Celtic families have been reported to be as high as 74% [9]. Also, in areas where caucasians have emigrated to, a high incidence can be found. On the other hand only few cases are reported among people from other genetic descent [8,13,15-19]. DD has a strong genetic predisposition and it is believed that the disease is inherited via an autosomal dominant gene with variable penetrance [20-22].

DD affects mainly patients older than 50 years [5-9,13,23]. This leads to an expected increase of incidence along with the steadily growing life expectancy [5-9].

A number of studies postulate that DD is linked with repetitive manual labour and the incidence is up to 3 times higher compared to patients without such history of manual labour [5,6,11,24,25]. Clinical experience suggests that the disease may start or the contracture may proceed, after trauma but no statistical evidence has been shown yet [25].

Lifestyle factors like smoking or heavy drinking are also believed to of risk for Dupuytren's disease (DD) [11,19,26-29]. Some diseases like diabetes [6,8,24,27,30,31], epilepsy or hypercholesterinemia have also been linked to DD but there is no clear evidence.

Conservative therapies have not been shown to be of substantial benefit, so that the most common treatment for DD remains the surgical excision of the diseased tissue and if necessary correction of finger contractures [6,32-36]. The gold standard of most operative techniques is a limited fasciectomy or total fasciectomy in some cases [32,37,38]. However, recurrence of the disease is high even after the complete palmar fascia has meticulously been excised [35].

Some complications are associated with operative treatment of DD such as nerv or vessel injuries, wound complications, skin necrosis or tendon injuries. Thus, the indication and timing of surgical intervention has been a subject of controversy [35,36].

Our institution was one of the first academic speciality centres for Hand Surgery in Germany and a considerable number of patients have been treated surgically within the last 50 years. We analysed our collective data of patients surgically treated for DD to evaluate the timing and indication for the necessity of surgical intervention. At the same time we wanted to achieve more insight into the coherence of the patients and their comorbidities or concomitant diseases, the severity of the disease and the statistical distribution of patients with regard to the affected hand and fingers in comparison to the so far published data in the literature.

## Methods

All available data from the medical records from all patients documented for (non outpatient) operative treatment of Dupuytren's disease (DD) in our Department from January 1^st ^1956 to August 15^th ^2006 were retrospectively analyzed (Table 1, and Table 2).

**Table 1:** Data of patients with surgery for DC between 1956 and 2006.

Acquired data from 1956–1988	
Parameter	Factors

age	age at operation in decades
Sex	male/female
affected hand	right/left/both
maximum degree per Hand	1°–4° after Iselin's classification
intraoperative complications	tendon-, nerv- or vessel-injury
postoperative complications	infection, skin necrosis, bleeding
other diseases	diabetes mellitus, epilepsy

**Table 2:** Additional parameter between 1988 and 2006.

Acquired data from 1988–2006	
Parameter	Factors

age	age at operation
Sex	male/female
affected hand	right/left/both
affected finger	digit 1–5
degree per finger	1°–4° after Iselin's classification
degree per Hand	sum of degree per affected finger
isolated or combined affection	one or more finger per hand
operation technique	limited or complete fasciectomy, skin transplantation
intraoperative complications	tendon-, nerv- or vessel-injury
postoperative complications	infection, skin necrosis, bleeding
other diseases	diabetes mellitus, epilepsy
simultaneous operation	carpal tunnel syndrome, trigger finger
lifestyle condition	alcohol, nicotin
recurrence of DD	yes/no

The degree of DD per finger is classified after Iselin [33] from stage one to stage four (Table 3). The Iselin's classification is the established staging method in our clinic. A retrospective modification to Tubiana's classification was not possible.

**Table 3:** Iselin's classification of Dupuytren's disease.

	Iselin's classification of different stages of Dupuytren's contracture
Stage 1	nodules or strands without affecting a finger joint
Stage 2	MCP joint affected
Stage 4	MCP and PIP joint affected
Stage 5	MCP and PIP joint affected with hyperextension in the DIP joint

To compare the hands and to show correlations between comorbidity and the stage of the disease we calculated a mean stage of affection per hand. The degree of DD per hand was calculated by the sum of the degree per affected fingers modified after Brenner et al[6] who used this classification combined with Tubiana's classification: (eg. digit 4: 3°, digit 5: 2° = 5 for the affected hand), meaning that a maximum value of 20 on this scale can possibly be achieved per hand.

All comparable data from the medical records of all patients between 1956 and 2006 were summarised in a descriptive statistic analysis using SPSS. Because there was no normal distribution in our data we used the Mann-Withney U-test for two unrelated variables and the Wilcoxen test for more unrelated variables. A p value of ≤ 0.05 was set to be significant.

## Results

Between 1956 and 2006 we operated 2919 patients for Dupuytren's disease (DD) of the hand in our Department (Table 4). 2579 (88.4%) were men and 340 (11.6%) were women (sex ratio men/women: 7,6:1).

**Table 4:** Data of all Patients between 1956 and 2006

Sex	men 2579		female 340
hand	right 834	left 616	both 1469
Complications	nerv injury 108 (3.7%)		tendon injury 5 (0.2%)
complications	skin necrosis 76 (2.6%)	infection 94 (3,2%)	bleeding 35 (1.2%)
diseases	diabetes mellitus 306 (10.5%)		epilepsy 39 (1.3%)

**Figure 1:**
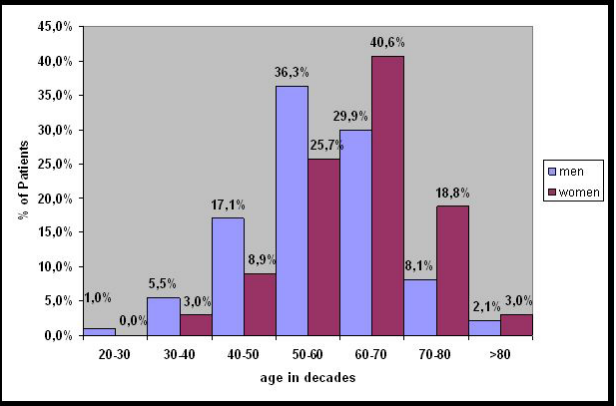
Percentage of all patients at time of operation classified in decades divided in men and women. In this figure it is obvious that operation time point in women is one decade later than in men.

79% of the Patients were between 40 and 70 years old (Figure 1).

**Figure 2:**
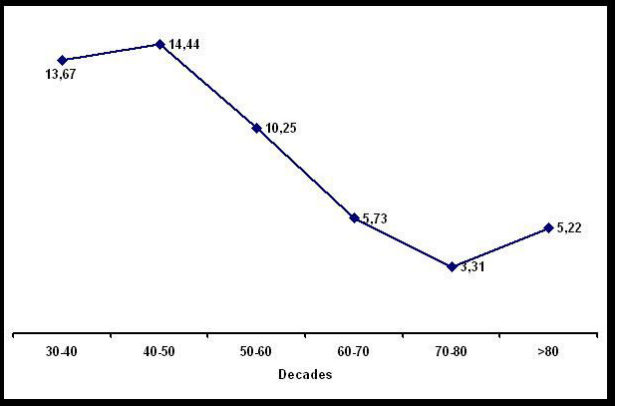
Sex ratio men to women classified in decades

The men/women ratio was indifferent in the different age groups but showed a decreasing tendency with increasing age of the Patients (Figure 2).

Half of the Patients which were operated on one hand were also affected by DD on the other Hand (50.3%). 27% of single affected hands were right and 22.6% were left hands.

Nerve injuries had been observed in 108 cases (3.7% of all operations).

The injury of a tendon was noted as a rare surgical complication in DD counting only 4 cases or 0.2% of all operations in which tendons were accidentally injured within the 50 year period.

In 1.2% of all operated hands a secondary bleeding was documented. Skin necrosis was documented in 2.6% of all operations.

269 patients or 9.2% of the whole collective were diagnosed with diabetes mellitus and 34 patients (1.2% of all patients) with epilepsy.

Wound infection was observed in 94 patients (3.2%)

### 1956–1988

**Figure 3:**
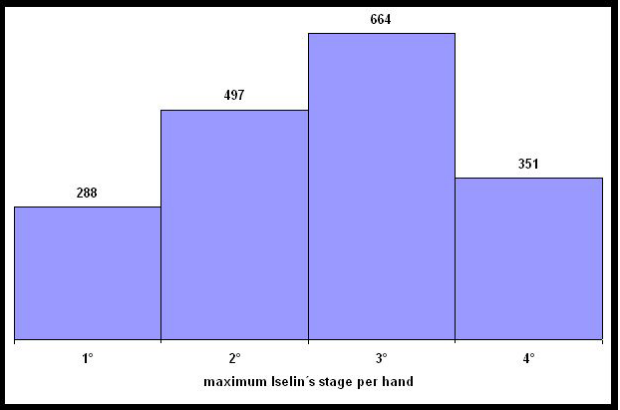
Maximum stage of Dupuytren's disease per hand between 1956 and 1987.

Because data from the historic medical records between 1956 and 1988 was not as consistent in all details as in the later period, the analysis of the degree of DD is demonstrated as maximum degree per finger per hand for this subgroup (Figure 3). 664 hands had at least one finger affected with 3°, 497 hands with stage 2 followed by stage 4 with 351 hands and at least 288 hands with stage 1.

### 1988–2006

We found 977 male and 142 female patients between 1988 and 2006 with a mean age of 57.62 years in the male collective and 62.62 years in the female collective (p > 0,001) (Table 5).

**Table 5:** Data of the patients from between 1988 and 2006.

age	men 57.62 ± 10,75	female 62.62 ± 10.55	
Mean stage	men 4.59 ± 2.54	female 4.26 ± 2.50 (p = 0.171)	
occurence	one finger 505	more finger 614	
therapy	limited fasciectomy 1061 (94.8%)	total fasciectomy 58 (5.2%)	
amputations	13 little fingers		
Lifestyle	smoking 185 (16.5%)	alcohol 236 (21.1%)	both 76 (6.8%)
simult. operation	carpaltunnel 14 (1.2%)	trigger finger 26 (2.3%)	

The mean stage of Dupuytren's disease (DD) per hand was 4.54 (women 4.26, men 4.59, p = 0,171).

**Figure 4:**
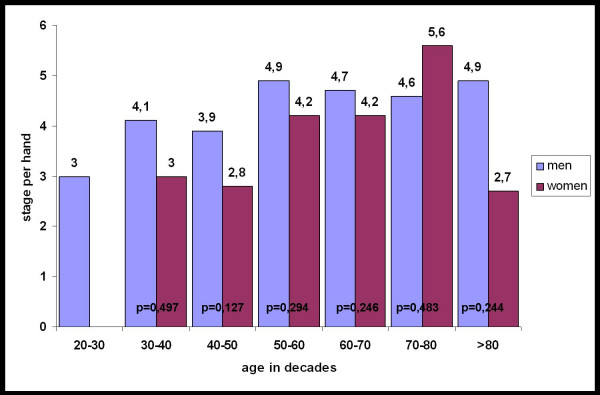
Mean stage of Dupuytren's disease in men and women classified in decades.

In 505 patients we found an isolated affection of one digit, in 614 patients more than one finger was affected. Within the age decades there were no significant differences between male and female concerning the mean degree per hand (Figure 4).

We observed a significantly increased stage per hand (p = 0,001) when both hands (mean stage 4.91) were affected compared to patients with just one hand affected (mean stage 4.32).

**Figure 5:**
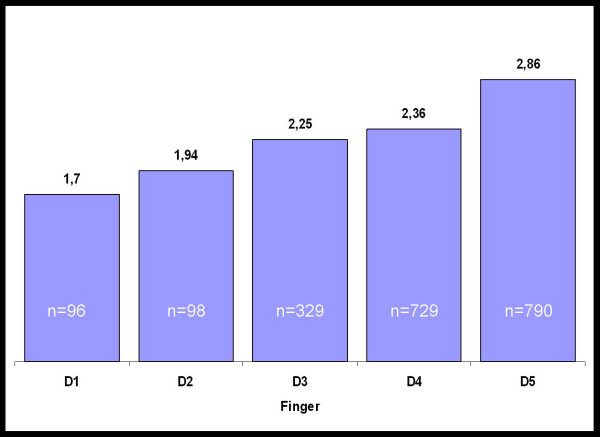
Mean stage of Dupuytren's disease per Finger in all Patients from the year 1988 to the year 2006.

In the group of 1119 operated patients we found 2042 affected fingers. The frequency of affected fingers increased from the radial to the ulnar aspect of the hand. We found 96 affected thumbs, but 790 affected little fingers (Figure 5). The mean stage of DD also increases from the radial to the ulnar aspect of the hand. While the mean stage of the thumb is 1.70° the little finger is affected by mean stage 2.86° (Figure 5). This observation is similar in men and women. There were no statistical differences between men and women in terms of the mean stage per finger except the middle finger (p = 0,009).

In 1061 cases limited fasciectomy was performed and in 58 cases total fasciectomy. In 13 cases an additional amputation (all were little fingers) was necessary because of a severe contracture between 1988 and 2002.

12% of all Patients were operated for a recurrent DD. All other patients were operated for the first time because of DD between 1988 and 2006 (Table 6). The mean stage per hand was not significantly different between recurrent and primary disease. However men tend to an increased stage while women tend to an decreased stage of Dupuytren's disease (DD) at the point of reoperation. In comparison to the whole collective and patients with primary DD the mean age between men and women was not significantly different in patients with recurrent DD. However patients with recurrent DD tend to be younger at the point of operation than patients which were operated because of primary DD.

**Table 6:** Recurrent Dupuytren's disease versus primary DD between 1988 and 2006

		Primary DD	Recurrent DD	p-value
number	men	857	120	X
	women	127	15	X
mean stage per hand	men	4,56	4,78	0,714
	women	4,26	4,20	0,178
	p-value	0,376	0,045	X
mean Age	men	57,87	55,89	0,187
	women	62,97	59,60	0,186
	p-value	>0,001	0,353	X

The mean stage per hand was calculated by the sum of the affected fingers, mean age in years

Diabetes was not significantly correlated to the stage of DD (p = 0,1333). Nevertheless, a slight tendency towards a lower stage was seen in patients with Diabetes mellitus at the time of operation. On the other hand a statistically significant increased stage was found in patients with epilepsy (p = 0,032).

The stage of DD per hand did not have any statistical effect on the number of intraoperative complications (p = 0,237). However, there was a correlation between the stage of DD and the rate of postoperative complications (p = 0,011).

16.5% of patients had consumed more than 20 cigarettes a day, 21.1% had consumed more than 40 g alcohol per day and 6.8% did both.

We found no statistical correlation between alcohol consumption (p = 0,248), smoking (p = 0,578) or smoking together with drinking (p = 0,079) on the stage of DD per hand in our collective.

Simultaneous operations for concomitant hand disorders were reported in a small number of patients (carpal tunnel syndrome 1.2%, trigger finger 2.3%).

## Discussion

Dupuytren's disease (DD) has been extensively investigated since its first description in the 16^th ^[2] and 17^th ^centuries. However, discussions on the exact cause of the disease continue.

In Germany about 1.9 Million people are affected by DD [6]. It is a common disease in North-western Europe and presents a great economic and surgical burden to our society [6,8,9,11,13,19,39]. The pathology of DD is well known. But neither the etiology nor the pathogenesis is completely understood. Published literature often refers to multi centre experiences and to data of non operated patients regarding incidence and prevalence of this entity.

Therefore after 50 years of experience with operative treatment of DD it seemed to be appropriate to compare the considerable amount of data to what has been published so far in smaller patient collectives.

To our knowledge this is the largest number of patients in a single series to be analyzed over period of 5 decades. In the present study a total amount of 2919 medical records of surgical treated patients was evaluated.

Other studies focus on prevalence, the clinical, serological and social assessment [9] or assumed risk factors for DD [9,12,23,26,27,30,31]. This study focused on the correlation of clinical appearance and the stage of the disease as well as on any detectable combination of typical patterns with other conditions. The type of operative treatment and the complications were evaluated in relation to the DD stages, as well.

A review of published data reveals that the prevalence of DD varies depending on the analyzed group of age, author and country [40,9,6].

In all these studies the prevalence in men is higher than in women depending to the age of the analyzed patients. With increasing age the prevalence of DD in men and women is adapting [6,39]. Our data is in concordance with this trend. However, Brenner et al. found a similar number of female and male patients in elderly patients [6].

In the present study we found a male to female ratio of 7,6:1 reflecting a typical value in the published range of other reports [6,10,39].

The mean age in men (62.6 years) at the time of operation was significantly higher than in women (57.6 years). These data slightly vary between other studies but almost all authors found a significant difference between men and women concerning mean age of prevalence or age when treated because of Dupuytren's disease (DD). Most of the male patients were operated in their sixties (36.3%) while the majority of women received surgical treatment in their seventies (40,6%). This compares favourably to other reports published so far [6-8,39,40].

To date it still remains unclear why men are affected more frequently and at younger age than women. Hankin et al. [41] tried to explain this phenomenon by the oestrogen and progesterone receptors in the palmar fascia but this has not bee proven so far.

As published previously[5,7,9,42] the mean stage of DD per hand in our study was not significantly different between male and female patients between 1988 and 2006 concerning all age groups. Neither were found significant differences comparing the mean DD stage of the right and the left hand [6]. However, we found a significantly higher stage per hand in patients with bilateral affection compared to patients with only one affected hand, so far without an explanation. Some authors have proposed that there is no influence of right- or left-handedness on the stage of DD. Millesi claims that the disease usually starts unilaterally and after sufficient time a bilateral manifestation can be seen [43]. This might be a possible explanation for our observation.

In the present study more right than left hands were affected. A possible reason for this might be that more people are right handed in Germany and patients rather consider the more important hand for operation because of their handicap in everyday life.

In contrast to other studies [6] we found 45.1% hands where only one digit was affected. Other authors have described single digit affection to be rare. Brenner et al reported single digit affection in 13% of their patients [6]. We believe this could be due to the philosophy of the surgeon at which stage Dupuytren's disease (DD) should be operated. This parameter might also represent a positive selection by the surgeon.

The mean stage per hand in our reviewed collective is 4.59° in male and 4.26° in female patients.

In most other studies Tubiana's classification was used to classify the stage of the disease. Due to the medical records it was not appropriate to change the traditional Iselin's classification used in our hospital into Tubiana's classification. So we think it is not useful to directly compare the data with other studies.

As a tendency we found an ulnar affection of the hand more often than a radial affection. This is in concordance to other published data regarding the pathology of DD [6,7,19,25,27,32,42]. While some authors found the fourth digit to be the most affected we discovered the fifth finger to be the most frequently affected digit [6]. The most likely combination of affected fingers in the present study is the fourth and fifth finger followed by the third and the fourth finger. Similar results were described by other authors [6,7,19,25,27,32,42]. Affection of the thumb and the index finger was found to be rare in our patients. In contrast to other studies we found more affected Index digits than thumbs. We cannot find a reason for this in our data but the differences compared to other studies are minimal.

The severity of the affected finger also increased from the radial to the ulnar aspect of the hand. An explanation for this phenomenon has not been found yet, but it might be speculated that embryological developmental phenomena afflict the anatomic structures involved. Because of its predominant ulnar affection of the hand DD has been linked to a potential lesion of the ulnar nerve [44], but this theory has not been evidenced yet. Further insights into the cellular or developmental background might be gained by cell culture experiments and experiences from tissue engineering results with fibroblasts, myoblasts and other cells in culture [48-54].

The common operative technique for Dupuytren's disease (DD) is a limited fasciectomy, total fasciectomy or occasionally simple fasciotomy [32,33,45].

**Figure 6:**
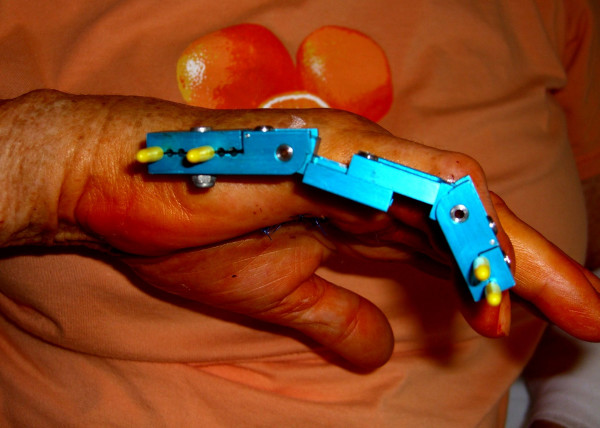
Erlangen external traction device to correct soft tissue and joint contracture before fasciectomy.

**Figure 7:**
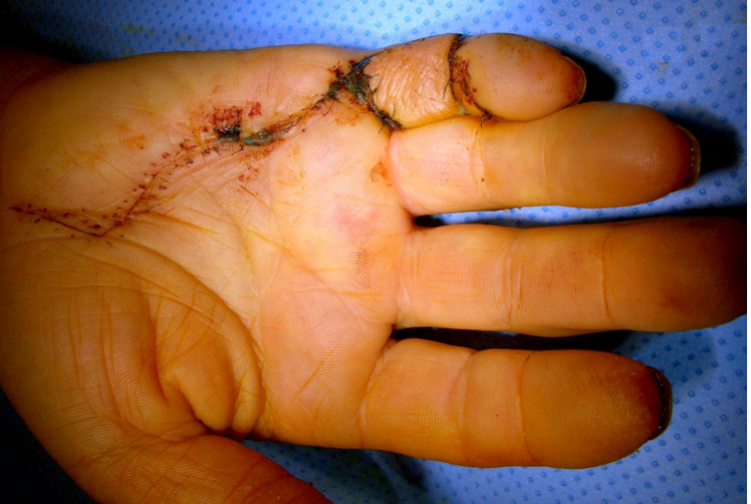
Patient with Cross-Finger-Flap from digit 4 to digit 5 to fill up a skin deficit after correction of the Dupuytren's contracture stage 4 where distraction was not possible.

In advanced cases an amputation may be necessary. Amputation is reserved only for cases without a surgical option for the contractures correction or for intraoperative ischemia when correcting the contracture without a previous stretching therapy. The rate of amputations was completely reduced after introduction of a distraction device in 2003. This device allows continuous elongation of the contracted soft tissue and distraction of the contracted joints prior to fasciectomy in 2003 (Figure 6). In addition we performed cross finger flaps in appropriate cases to fill the skin defects when sever 4^th ^degree contraction demanded additional soft tissue cover and distraction therapy was not an option (Figure 7).

The intra- and postoperative complication rate in our whole collective was very low. These results are similar to so far published data [7,13,32]. In our opinion the complication rate depends on operation technique. Some authors found an increased complication rate in total fascectomy compared to limited fasciectomy[33]. In our hospital limited fasciectomy is traditionally favoured (94.8% of the operations).

Recurrence rate in Dupuytren's surgery is described to be 26% to 80% depending to the study. Astonishingly we only found 12% of patients being operated for a recurrent DD. However, this confirms our clinical experience that about every tenth DD operation is for recurrent disease. The tendency in our data that patients with a recurrent disease are younger might be explained by the confirmed data that younger patients suffer from a more aggressive form of DD. As we found in the whole collective and in patients with primary disease the mean stage per hand is decreased in women compared to men.

Some comorbidities or lifestyle-conditions are believed to influence the prevalence of Dupuytren's disease (DD) [26,28,29,31]. It is a common opinion that DD is strongly related with specific disease such as diabetes [12,24,27,30,46], liver fibrosis, and epilepsy [8,24,27]. The prevalence of diabetes in our collective was 10.5%, being slightly higher than the all over prevalence of diabetes mellitus in Germany in 2006 which is 6.9%. Other authors found similar or lower results [6]. Some authors found a lower stage of DD in patients with diabetes mellitus compared to other patients, which we were able to confirm in the present study [8,24,30,47,48], indicating that patients who suffer from Diabetes mellitus might have a milder progression of DD. Only one study found diabetes mellitus to be a strong risk-factor for DD [46].

The prevalence of epilepsy in our patients (1.3%) also is increased compared to the all over prevalence of epilepsy in Germany (0,5% – 1%). But it is unclear if epilepsy itself or the medication (Phenytoin) is the risk factor for the development of DD.

With 16.5% patients who smoked more than 20 cigarettes a day the rate is rather low compared to other publications. However, other authors do not state how many cigarettes the patients smoke per day and include all smokers in this group. Smoking may be a risk factor for DD because of the altered microcirculation and the generation of free oxygen radicals [12]. Drinking is also believed to be a risk factor for DD for the same reason. In our collective from 1988 to 2006 we found 21.1% of patients who consumed more than 40 g alcohol per day, while data from other authors are indifferent. This may be due to different inclusion criteria regarding the values of alcohol consumption or because of different drinking habits of the analyzed patient collective. 6.8% of our patients reported an uptake of more than 40 g alcohol and smoked more than 20 cigarettes a day. Significant differences on the mean stage per hand in these patients were not detected.

## Conclusion

While the etiology and pathogenesis of Dupuytren's disease (DD) remains unclear until today the present study supports well known facts and theories about DD whereas in this large cohort for the first time often claimed typical correlations of this entity with diabetes and alcohol consumption in this large single centre experience could not be verified.

## Competing interests

The author(s) declare that they have no competing interests.

## Authors' contributions

B L and V P carried out the study of the patient's record charts, and drafted the manuscript and performed the statistical analysis of data. R E H conceived and designed the study, wrote the final manuscript version. All authors read and approved the final manuscript.

## Pre-publication history

The pre-publication history for this paper can be accessed here:


